# Summer Diarrheas of Children and Their Treatment

**Published:** 1902-09

**Authors:** M. A. Auerbach

**Affiliations:** New York City, Medical Inspector, Department of Health


### SUMMER DIARRHEAS OF CHILDREN AND THEIR TREATMENT.

BY M. A. AUERBACH, PH.G., M.D., NEW YORK CITY,
Medical Inspector, Department of Health.

The importance of these demand a separate consideration. Three forms, more or less distinct, can be recognized, viz., acute dyspeptic diarrhea, cholera infantum, and acute entero-colitis.

### ACUTE DYSPEPTIC DIARRHEA.

This disease is chiefly due to errors in diet, which do not necessarily consist in the substitution of unnatural foods for the mother's milk. The mother's milk may be altered in quality by emotional causes, by improper food and improper hygiene. Or it may be caused by overfrequent nursing. More often, however, it is caused by the ingestion of unnatural foods.

There are also predisposing influences which facilitate the action of the exciting causes. These are especially dentition and the extreme heat of summer.

*The prognosis* of the aforesaid disease among the better classes is commonly favorable, but among the weak, puny, and half-starved children of our lower east side large numbers perish, especially during the summer months.

*The old-time treatment* in these cases was a primary purge, calcined magnesia, or castor oil. After the purge bismuth sub-nitrate or prepared chalk was given. Since the introduction of Glyco-Thymoline (Kress) the above-mentioned methods have been cast aside. A very good and effective prescription, which has given me most splendid results in these kind of cases, in conjunction with a carefully restricted diet, is,

| | | |
|---|---|---|
| ℞ | Bismuth sub-nitrate | Dr. I |
| | Tr. opii deoderatum | M. X |
| | Glyco-Thymoline | Oz. II |
| | Aqua rosarum ad. q. s | Oz. IV |

Misce et Sig. Dr. 1 every 3 hours for a child one year of age.

### CHOLERA INFANTUM.

A variety of acute catarrhal enteritis of intense severity, corresponding in symptoms and course to cholera morbus in the adult, but much more serious in termination.

*Prognosis* in these cases is at best not very favorable, although recovery is not impossible.

*Treatment* of these cases is of quite a different nature from those above mentioned. In the first place, the fever must be combated and I know of no better method than a bath containing some Glyco-Thymoline, at about 80° F., reduced by adding small pieces of ice to 70° or 65°. Next pain, to reduce pain 100th of a grain of morphine sulphate can be administered to a child of one year. Stimulation with strychnia hypodermically, iced champagne to prevent vomiting, brandy, whisky and other stimulants.

One of the best methods for irrigating the large intestine is by introducing a small soft catheter through the rectum and injecting into the bowel about a pint to a pint and a half of warm water containing about twenty-five per cent. of Glyco-Thymoline. This I find removes and prevents the reaccumulation of the fermentative as well as the putrefactive products of the bowel. Should, however, the hyperpyrexia continue, the douche may be given at a lower temperature. During convalescence great care must be taken in the feeding of the patient.

ACUTE ENTERO-COLITIS

is an affection of inflammatory nature more severe than dyspeptic enteritis, chiefly of the ileum and colon, affecting especially the lymph follicles. This, like the preceding, is a disease of the hot months of summer, and the period of teething especially. It is produced by the same causes as dyspeptic diarrhea. It is most frequent during the ages of six and eighteen months. It likewise may be a termination of dyspeptic diarrhea or of cholera infantum.

*Treatment.*—The general surroundings and hygiene necessarily play an important part. The medical treatment, however, is somewhat different. Anodynes are more imperatively demanded because there is greater suffering, and depletion may be needed in the beginning by salines, though good judgment is required because the child's strength must be watched. The colon should be flushed with a solution of Glyco-Thymoline having a strength of twenty-five per cent. This I find answers admirably in these cases. The solution may be made with iced water. The coming teeth should likewise be watched and the gums be scarified whenever required.

I will supplement my remarks by adding a few of the many cases treated with Glyco-Thymoline and leave results to speak for themselves.

CASE 1.—M. K., aged eight months, male, was taken with severe vomiting and colicky pains at night. The vomita contained lumps of coagulated milk. The stools were very offensive and recurred at intervals of tweny minutes. I left a prescription for Glyco-Thymoline, two ounces; bismuth sub-nitrate, one drachm; rose water, enough to make four ounces. I called the next morning and found but little improvement in my patient, and at once flushed out the bowel with a 25 per cent. aqueous solution of Glyco-Thymoline and continued the prescription given the night previous. This treatment was continued for three days, the patient steadily improving during that time. I recommended that the child be taken away from the city, which was done. I heard later from the parents that the child had not had a relapse, but made a speedy recovery.

CASE 2.—Mary C., aged seven months and a half, was brought to my office, her little knees drawn up, a look of anguish on her face, which was pale and drawn, with eyes protruding. She had a number of watery discharges from the bowels, incessant vomiting, a temperature of 103½, a rapid and feeble heart. A further examination of this poor little tot was unnecessary. Anodynes were at once administered to soothe the pain. I washed out the bowels with a 40 per cent. solution of Glyco-Thymoline and administered the same in a 50 per cent. solution with peppermint water internally in doses of one teaspoonful repeated every two hours. The results in this case far exceeded my expectations. The child made a slow but successful recovery.

CASE 3.—L. P., male child, aged sixteen months; was called to check the diarrhea, which was of a serious character, having a tinge of blood. The vomiting was not of a severe nature, the only alarming symptoms the child showed were the intestinal ones. Three separate washings of the child's colon were made at intervals of six hours. The child's food was restricted to barley water; this case, like the one preceding, made a perfect and speedy recovery.

CASE 4.—R. A., a little tot of the East Side, aged thirteen months, brought up in one of the dark and dingy rooms of a tenement house; this poor little one was suffering for five days before my attention was called to the case. I found it in an emaciated condition, unable to move a limb, the bowel movements were frequent and watery; the little one was on the point of collapse; strychnia was administered hypodermically to stimulate the heart; after which diluted brandy was given every half-hour. The colon was irrigated with twenty-four ounces of a 50 per cent. aqueous solution of Glyco-Thymoline; I had the child under my observation for two and one-half weeks, and with proper food and fresh air the child made a good recovery.

CASE 5.—M. M., a boy baby seven month of age, teething and causing all sorts ef trouble for its parents, who were well-to-do. Was summoned to the house early one morning, found the little one vomiting quantities of curdled milk, and movements having a decidedly fetid odor. I tried most everything in this case and received but small relief by the use of Glyco-Thymoline. Upon careful investigation, I found that the teeth were causing the trouble; the gums were then lanced, and the child's diet restricted; that is the breast feedings were given at three-hour intervals, and only lasting five minutes at a time. Glyco-Thymoline was kept up. With perseverance and good nursing our little one soon got well.

---

UP to June 29, 1740 cases of cholera, with 1385 deaths, occured in Manila, while 9444 cases with 7038 deaths developed in the provinces. Lieutenant-Colonel Maus says that there have been probably 2000 more deaths in the provinces, records of which have never been kept. The natives, according to the *Philadelphia Medical Journal,* tried to conceal the existence of cholera, and on that account absolute authority has been given the medical officers of the army. In the city of Manila, containing over 300,000 inhabitants, the deaths in any one day have not exceeded thirty, while in the provinces the death-rate has been double this. Quarantine regulations now make it almost impossible to get in or out of Manila. Up to May 12, the provincial medical board had succeeded in keeping cholera out of the provinces of Albay, a strict shotgun quarantine having been adopted. Though the cholera has twice appeared on shipboard in ports of the province, no communication with the shore was permitted. In Colonel Maus's last report, dated May 15, the death-rate was 6.01 among the soldiers—less than for any month on record.

